# Predictors of Complementary and Alternative Medicine Use in Cancer Care: Results of a Nationwide Multicenter Survey in Korea

**DOI:** 10.1155/2012/212386

**Published:** 2012-12-24

**Authors:** Ji-Yeon Shin, So Young Kim, Boyoung Park, Jae-Hyun Park, Jin Young Choi, Hong Gwan Seo, Jong-Hyock Park

**Affiliations:** ^1^National Cancer Control Institute, National Cancer Center, Goyang, 323 Ilsan-ro, Ilsandong-Gu, Goyang-si, Gyeonggi-do 410-769, Republic of Korea; ^2^Department of Social and Preventive Medicine, Sungkyunkwan University School of Medicine, 2066 Seobu-ro, Jangan-gu, Suwon-si 440-746, Republic of Korea

## Abstract

*Background*. Although studies have shown that the use of complementary and alternative medicine (CAM) is common in cancer patients, few surveys have assessed CAM use and associated factors in various cancers in Korea. *Objectives*. We explored factors predicting CAM use among a nationally representative sample of cancer patients. *Methods*. In total, 2,661 cancer patients were administered questionnaires about their CAM use and factors that might predict CAM use including sociodemographics, clinical and quality-of-life factors, time since diagnosis, trust in physicians, trust in hospitals, satisfaction, and informational needs. Data were analyzed using Pearson's *χ* ^2^ tests and multivariate logistic regression analysis. *Results*. Overall, 25.5% reported that they had used or were using CAM. Higher income, presence of metastasis, longer time since diagnosis, less trust in hospitals, lower overall satisfaction, and higher degree of informational need were significantly associated with CAM use. *Conclusions*. The use of CAM in patients with cancer can be interpreted as an attempt to explore all possible options, expression of an active coping style, or expression of unmet needs in the cancer care continuum. Physicians need to openly discuss the use of CAM with their patients and identify whether they have other unmet supportive needs.

## 1. Introduction

The National Center for Complementary and Alternative Medicine (NCCAM) defines complementary and alternative medicine (CAM) as “diverse medical and healthcare systems, practices and products that are not generally considered a part of conventional medicine” [1]. Despite advances in the medical treatment of cancer that have resulted in improved cure rates, the incidence of cancer is increasing, and it remains a leading cause of death [2]. At the same time, there is an increasing tendency for patients with cancer to use CAM by [3]. In patients with breast cancer, the rate of CAM use increased from 67% to 82% between 1998 and 2005 [4]. Furthermore, CAM use was reported in up to 90% of patients with cancer in the United States [5, 6] and 44.6% of those in Japan [7].

From a clinical perspective, it is important to identify factors that encourage large numbers of patients with cancer to use CAM, solely or concomitantly with conventional medical treatments, because cancer patients frequently face situations that are subjectively less controllable and more frightening than other chronic or life-threatening diseases [8]. Previous studies have suggested that the rate of CAM use depends on sociodemographic characteristics of patients, cancer-related clinical characteristics, regional and cultural factors, and patients' patterns of coping with the disease [9]. Younger age [7, 10], female gender [10, 11], a higher level of education [7, 10, 12], and higher income and social class [10, 11] seem to be associated with more frequent use of CAM. Additionally, it has been reported that a higher rate of CAM use is associated with a longer duration since diagnosis [13], progression of the cancer [10, 12, 14], and a lower degree of trust in physicians [10, 15].

Meanwhile, few studies have been conducted to examine the relationship between the unmet informational needs and the use of CAM in patients with cancer, although the topic of unmet needs has been a recurrent on in CAM-related studies [10]. In patients with cancer, informational support provides a sense of control over the illness [16] and enhances patients' quality of life when information meets their needs [17]. Accordingly, unmet needs for information may cause distress in patients [18], thus possibly affecting their pattern of use of medical services. It is thus important that the degree of patient need for information is examined as a possible predictor of CAM use.

In Korea to date, almost no nationwide studies have been reported examining the use of CAM and relevant factors. Most previous studies have been conducted at a few hospitals in a specific region. The rate of CAM use varies depending on the study; it was reported to be 63.7% by Kim [19], 55.5% by Lee et al. [20], and 56.9% by Park and Lee [21]. It is worthwhile to examine the current status of CAM use in patients with cancer and to develop the best policy for them. To do this, it is important to collect baseline data that can be generalized for various types of cancer in varying stages. This should also be accompanied by access to comprehensive cancer care to achieve a better understanding of the factors affecting CAM use and relevant reasons.

Given this background, we conducted this study with the following objectives: (1) to evaluate the status of CAM use based on nationwide data in patients with various types of cancer; (2) to examine factors affecting CAM use, including socioeconomic, clinical, and individual factors, in patients with cancer; and (3) to identify any association between informational needs and CAM use in patients with cancer.

## 2. Materials and Methods

### 2.1. Study Patients and Procedure

For the current study, we collected data from patients with cancer who were treated at the National Cancer Center or nine regional cancer centers in Korea during the period from July to August 2008. Quota sampling was used: 80% of the patients had been diagnosed with one of six major types of cancer (stomach, lung, liver, colon and rectum, breast, and cervix), and the remaining 20% had other types of cancer. The patients were interviewed by trained interviewers at their treatment centers. Inclusion criteria were (1) age >18 years old, (2) an established diagnosis of cancer, (3) a period >4 months since the diagnosis, (4) current treatments or follow-ups, and (5) written informed consent for study participation. Detailed procedures have been described elsewhere [22]. In total, 2,661 patients with cancer completed an interview. Then, through a retrospective analysis of the patients' medical records, we obtained clinical data such as types, histology, and SEER stage of the cancers. The current study was approved by the institutional review board (IRB) of the National Cancer Center in Korea.

### 2.2. Measurement and Analysis of the Patient Data

A questionnaire survey was designed to collect data about CAM use and potential predictors (sociodemographic and clinical factors, quality of life, the degree of trust in physicians and hospitals, degree of satisfaction with conventional medicine, and degree of need for information).

We defined CAM based on the widely established NCCAM taxonomy [1] and included alternative medical systems, mind and body interventions, natural products, manipulative and body-based methods, and energy therapies. The specific CAM modalities asked about in this study were traditional Chinese medicine, ayurveda, herbalism, acupuncture, meditation, yoga, biofeedback, hypnosis, relaxation, imagery, prayer, chiropractic, massage, special diets, dietary supplements, qi gong, and healing touch. According to NCCAM, we did not include pure psychotherapeutics or support groups, and we excluded general exercises to avoid overestimation.

In the questionnaire survey, patients were asked about their experience using CAM with the question, “Have you ever used complementary and alternative medicines (CAM) since you were diagnosed with cancer?” Patient responses were collected as a dichotomized variable. Sociodemographic factors included age, gender, education, income, marital status, and health insurance status. Clinical factors included the type of cancer, SEER stage, treatment, time since the diagnosis, and current disease status. Additionally, quality of life was measured using the EQ5D, which measures five dimensions of patient quality of life (mobility, self-care, usual activities, pain/discomfort, and anxiety/depression) using a 3-point scoring system (1 = no problem, 2 = some problems, and 3 = severe problems). The EQ5D has been validated for Korean subjects [23]. We classified responses to the EQ5D into two categories: patients who had at least one problem and those who had no problem.

The degree of trust in physicians and hospital was measured using a 5-point Likert scale (1 = strongly disagree and 5 = strongly agree), and responses were classified into three categories: very satisfied, somewhat satisfied, and not satisfied.

The overall degree of satisfaction with conventional cancer care was measured with a 5-point Likert scale (1 = very dissatisfied and 5 = very satisfied), and responses were then dichotomized (satisfied or very satisfied versus others) for further analyses.

The degree of patient need for information was measured using a subscale of the Comprehensive Needs Assessment Tool (CNAT) in cancer, which has been validated for Korean subjects [24]. Patients were instructed to evaluate items on a 4-point scale (never needed, slightly needed, moderately needed, and highly needed) based on the past month's experience. Responses were then classified for further analysis into binary variables: “never needed” versus “needed” (slightly needed, moderately needed, or highly needed).

### 2.3. Statistical Analysis

We used the chi-squared test to analyze differences in the rates of CAM use between the categories of selected demographic and clinical variables (age, gender, education, marital status, monthly income, national health insurance, cancer type, metastasis, SEER stage, and time since diagnosis). Additionally, we performed a univariate analysis of factors predicting CAM use. Then, we entered variables found to be significantly associated with CAM use in the univariate analysis into a multiple logistic regression model. The criterion for variable entry was *P* = 0.05. Age, gender, education, monthly income, cancer type, metastasis, and time since diagnosis were included in a basic predictive model. EQ5D, trust in physicians and hospitals, overall degree of satisfaction with the conventional cancer care, and degree of patient need for information were also analyzed individually for their associations with the use of CAM following adjustment of variables in the basic model. Data from patients with missing values were excluded from the multiple logistic regression model. All statistical analyses were performed using the SAS 9.2 software (SAS Institute, Cary, NC, USA). Statistical significance was set at *P* < 0.05.

## 3. Results

Table 1 summarizes baseline characteristics of all study patients, including those who used CAM. Of the 2,661 total respondents, 25.5% responded that they had used CAM. The rate of CAM use was relatively higher in women, the age group between 40 and 59 years, and the group with monthly household income over USD 3000. With regard to type of cancer, CAM use was relatively higher in patients with breast cancer. It was also relatively higher in patients who had metastasis, those with a longer duration since diagnosis, those who had a lower degree of trust in physicians or hospitals, and those who had a lower overall degree of satisfaction with the cancer treatment offered by hospitals. Furthermore, from the perspective of quality of life, the rate of CAM use was relatively higher in patients who responded that they had a problem in more than one area covered by the five EQ5D questions.

A univariate analysis was performed for the variables presented in Table 1. This was followed by a multivariate analysis of variables showing a significant association with CAM use. The basic model included variables of gender, age, education, monthly income, cancer type, metastasis, and timing of diagnosis. Variables showing a significant association with CAM use in the univariate analysis, including gender, age, and education, no longer showed significant associations or showed a lower degree of association following adjustment for other variables. However, monthly income, cancer type, metastasis, and the duration since diagnosis maintained significant associations with CAM use even following the adjustment for other variables. Next, we performed a multivariate logistic regression analysis including trust in physicians and hospitals, overall degree of satisfaction with conventional medical service, and the EQ5D scores individually in a basic model. Even after controlling for socioeconomic and clinical factors, lower degree of trust in physicians or hospitals (aOR 2.38, 95% CI 1.13–5.00; aOR 2.04, 95% CI 1.15–3.63, resp.), and lower overall degree of satisfaction with the hospital (aOR 1.61, 95% CI 1.11–2.34) were significantly associated with the use of CAM. With regard to EQ5D, the patients who responded that they had a problem in one or more of the areas addressed in the EQ5D were 1.23 times (95% CI 1.01–1.50) more likely to use CAM compared with those who responded that they had no problems (Table 2).

The analysis of the association between the use of CAM and the degree of patient need for information indicated that CAM use was predicted by higher degree of informational need. Patients who responded that they were in need of information about the status and further course of the disease, the test and treatment methods, correct diet, and availability of financial support for patients with cancer were more likely to use CAM. Of the nine questions related to this issue, only the question about hospice showed no significant association with the use of CAM (Table 3).

## 4. Discussion

We conducted the current multicenter study to extensively analyze factors affecting the rate of CAM use in patients with various cancers in varying stages. Our results showed that sociodemographic factors affected the use of CAM, consistent with previous studies [7, 10–12]. A multivariate analysis showed that such sociodemographic factors as younger age (but not the youngest) and high monthly income had a significant association with the rate of CAM use. A higher level of education was also a marginal predictor of CAM use. Moreover, clinical factors, including metastasis, primary cancer site, and time since diagnosis, were also significantly associated with the use of CAM even after adjustment for sociodemographic factors. The result that metastatic cancer patients were more likely to use CAM suggests that patients at an advanced stage tend to use every method available to supplement conventional medicine. The association between progression of cancer and CAM use has been reported previously [10, 12, 14]. According to Paltiel et al., a poor progress may lead to increased level of concern or distress and a desire to explore all possible options. Our results also showed that the rate of CAM use was relatively higher in patients with breast cancer, also in agreement with previous reports [25–27]. It has been reported worldwide that the rate of CAM use is highest in patients with breast cancer [26]. This finding may be confounded by the fact that more women use CAM than men [28] and should be interpreted cautiously. Gender was included as a variable in a multivariate analysis, which might dilute the association between gender and use of CAM in patients with breast cancer. On the other hand, it is also probable that the gender-related difference in the rate of CAM use might be diluted following the adjustment for breast cancer. Our results might be interpreted in the latter manner.

After controlling for basic factors in the multivariate analysis, the degree of trust in physicians and hospitals was strongly associated with the use of CAM. A lower degree of trust in physicians was associated with more frequent use of CAM, and the same was also true of the association between the degree of trust in hospitals and CAM use. This is supported by several studies. According to Cassileth et al., doctor-patient relationships were worse in patients who underwent both conventional therapy and CAM compared with patients who underwent conventional therapy alone [15]. Additionally, Paltiel et al. reported that the rate of CAM use was relatively lower in patients who had a higher degree of trust in physicians [10]. 

Our results showed that overall degree of satisfaction with conventional medicine was also a predictor of CAM use. However, this remains controversial. It has been reported that patients tend to use CAM unless they are satisfied with conventional medicine [10, 29]. Thus, many oncologists worry that patients would discontinue current treatments with the purposes of using CAM alternatively to conventional medicine. On the other hand, there are also contradictory results that dissatisfaction with conventional treatment was not a predictor of CAM use [30], and patients who used CAM expressed as high a degree of trust in conventional medicine and showed as high a degree of compliance as patients who were not interested in CAM [9]. Further studies are needed to clarify the relationship between satisfaction with conventional medicine and CAM use conclusively.

Interestingly, patients who had a greater need for information were more likely to use CAM. According to the questionnaire survey, based on the responses to the nine questions about the degree of patient need for information, other than the question about hospice, a higher degree of informational need was associated with a higher rate of CAM use. These results can be interpreted from two perspectives. (1) A coping style might be involved. That is, patients tend to consider the use of CAM as a way of independently contributing to their treatment if they have an active coping style against the disease and consider their prognosis somewhat hopeful (and their need for information about hospice is accordingly lower). Söllner et al. reported that a coping style characterized by information seeking and active problem solving was a powerful independent predictor of CAM use [9]. (2) The correlation between CAM use and the degree of informational needs may be considered from the perspective of unmet needs. The concept of unmet needs in cancer patients comprises several aspects including information, psychological problems, physical symptoms, and emotional needs [31]. If patients with cancer have unmet needs that include informational aspects, this would tend to decrease the degree of satisfaction with conventional treatment and/or the trust in physicians or hospitals, which might in turn increase the rate of CAM use. It has been reported that patient satisfaction with medical treatments is increased when patients are appropriately provided with information [32]. Following the analysis of data obtained in the current study, a higher degree of informational need was correlated with lower overall satisfaction with conventional medical services and trust in hospitals and a lower degree of trust in physicians or hospitals (data not shown). Due to limitations of this cross-sectional study, however, it was difficult to identify any causal relationship between the variables.

In our clinical series, the overall rate of CAM use following the diagnosis of cancer was 25.5%. This is notably lower than previously reported: 44.6% in Japan [7], 61.5% in Australia [12], 51.2% in Israel [10], and 78–90% in the United States [3, 5, 6, 33]. Moreover, it is quite different from the 55–65% in previous Korean reports [19–21]. One possible explanation for this low rate is a slight difference in the definition of CAM between this and the previous studies. Although many studies [3, 5, 6, 12, 19–21] defined CAM based on the NCCAM, some [7, 33] used operational definitions of their own, and one study was based on a Cochrane collaboration [10]. Moreover, even if based on NCCAM, the specific modalities of each CAM category listed in the questionnaires varied among the studies. For example, our study excluded general physical exercises, while yoga and qi gong were included in the exercises as CAM. However, many studies did not distinguish between movement therapies and general physical exercises [3, 6, 12, 19–21]. Similarly, the exclusion of support groups and psychotherapy from our definition of CAM may have influenced the result. Another possible explanation for the rate differences is the questionnaire survey method. In studies that reported a high rate of CAM use, the questionnaire was self-administered by patients and returned by mail directly to the researchers [3, 5–7]. In our study, however, face-to-face interviews were performed by trained interviewers while patients were awaiting treatment. Since some patients felt uncomfortable about disclosing their use of CAM to their physicians, patients who completed a questionnaire survey right before (or after) seeing a doctor in the hospital might have a higher possibility of nondisclosure than those who completed it in their own homes. Consequently, our survey presumably underestimates the actual level of CAM use.

The limitations of the study are as follows. First, the current study is a cross-sectional one. Thus, there are difficulties in identifying any causal relationships between variables. Based solely on our results, it remains unclear whether the degree of patient satisfaction with conventional treatment was decreased because they used CAM or whether patients with a lower degree of satisfaction with conventional treatment tend to use CAM. Second, as mentioned earlier, we cannot completely rule out the possibility that our results might be an underestimate, although there were differences in the definition of CAM between this and the previous studies. This underestimate may affect the whole study. Continued research on the prevalence and predictors of CAM are needed in Korea. Despite these limitations, our results are of significance in that we extensively examined the status of CAM use and relevant factors in patients using a large sample size and various cancers in varying stages.

In conclusion, the use of CAM in patients with cancer can be interpreted as an attempt to explore all possible options, an expression of an active coping style, or an expression of unmet needs in the cancer care continuum. In any case, physicians should openly address the use of CAM and should identify whether there are other unmet supportive needs with their patients. Additionally, there should be sufficient communication between physicians and patients, which is essential for forming a trusting physician–patient relationship.

## Figures and Tables

**Table 1:** Characteristics of cancer patients and complementary and alternative medicine use.

Variables	Number of patients	Number of users	%	*P* (*X* ^2^ test)
Total	2661	678	25.5	

Age (year)				
18–39	185	50	27.0	
40–59	1171	355	30.3	
60–69	791	176	22.3	
70+	514	97	18.9	<0.001
Gender				
Male	1412	316	22.4	
Female	1249	362	29.0	<0.001
Education				
≤Middle school	1378	295	21.4	
High school	836	249	29.8	
≥College	436	130	29.8	<0.001
Marital status				
Married	2212	577	26.1	
Not married (single, divorced, and widowed)	446	100	22.4	0.105
Monthly household income				
<1000 USD	803	155	19.3	
1000–3000 USD	1138	300	26.4	
≥3000 USD	705	218	30.9	<0.001
National Health Insurance				
National Health Insurance	2297	594	25.9	
Medicaid/none/others	364	84	23.0	0.137
Cancer type				
Stomach	464	103	22.2	
Lung	322	77	23.9	
Liver	254	70	27.6	
Colon/rectum	340	64	18.8	
Breast	379	146	38.5	
Cervix	113	31	27.4	
Others	789	187	23.7	<0.001
Metastasis				
No	1901	438	23.0	
Yes	637	202	31.7	<0.001
SEER stage				
In situ and local	1038	251	24.2	
Regional	918	235	25.6	
Distant	468	125	26.7	
Unknown	237	67	28.3	0.517
Time since diagnosis				
≤12 months	985	204	20.7	
12–36 months	909	234	25.7	
36–60 months	385	120	31.2	
>60 months	382	120	31.4	<0.001
Trust in doctor				
Very	2335	575	24.6	
Somewhat	292	88	31.1	
Not at all	34	15	44.1	0.005
Trust in hospital				
Very	2125	514	24.2	
Somewhat	480	141	29.4	
Not at all	56	23	41.1	0.002
Satisfaction with medical service				
Very	1837	439	23.9	
Somewhat	673	185	27.5	
Not at all	151	54	35.8	0.002
EQ5D				
No problem	987	223	22.6	
One or more problem	1674	455	27.2	0.009

**Table 2:** Predictors of complementary and alternative medicine use.

Variables	Univariate analysis		Multivariate analysis	
	OR	95% CI	OR	95% CI
Basic model (*n* = 2515)				
Age (year)				
18–39	1.00	Reference	1.00	Reference
40–59	1.18	0.83–1.66	1.36	0.93–1.99
60–69	0.77	0.54–1.11	1.18	0.78–1.80
70+	0.63	0.42–0.93	1.21	0.71–1.77
Gender				
Male	1.00	Reference	1.00	Reference
Female	1.42	1.19–1.69	1.17	0.93–1.48
Education				
≤Middle school	1.00	Reference	1.00	Reference
High school	1.56	1.28–1.90	1.31	1.05–1.65
≥College	1.56	1.22–1.99	1.26	0.94–1.70
Monthly household income				
<1000 USD	1.00	Reference	1.00	Reference
1000–3000 USD	1.50	1.20–1.86	1.34	1.05–1.72
≥3000 USD	1.87	1.48–2.37	1.57	1.18–2.10
Cancer type				
Stomach	1.00	Reference	1.00	Reference
Lung	1.10	0.79–1.54	1.20	0.84–1.72
Liver	1.33	0.94–1.90	1.25	0.86–1.83
Colon/rectum	0.81	0.57–1.15	0.77	0.53–1.11
Breast	2.20	1.63–2.97	1.79	1.24–2.56
Cervix	1.33	0.83–2.12	1.05	0.63–1.77
Others	1.09	0.83–1.43	1.05	0.78–1.41
Metastasis				
No	1.00	Reference	1.00	Reference
Yes	1.55	1.27–1.89	1.55	1.26–1.91
Time since diagnosis				
≤12 months	1.00	Reference	1.00	Reference
12–36 months	1.33	1.07–1.64	1.32	1.05–1.65
36–60 months	1.73	1.33–2.26	1.79	1.35–2.38
>60 months	1.75	1.35–2.29	1.76	1.33–2.34

Extended model* 1 (*n* = 2515)				
Trust in doctor				
Very	1.00	Reference	1.00	Reference
Somewhat	1.32	1.01–1.73	1.26	0.94–1.67
Not at all	2.42	1.22–4.79	2.38	1.13–5.00
Extended model 2 (*n* = 2515)				
Trust in hospital				
Very	1.00	Reference	1.00	Reference
Somewhat	1.30	1.05–1.63	1.18	0.93–1.49
Not at all	2.19	1.27–3.76	2.04	1.15–3.63
Extended model 3 (*n* = 2515)				
Satisfaction with medical service				
Very	1.00	Reference	1.00	Reference
Somewhat	1.21	1.99–1.78	1.14	0.92–1.41
Not at all	1.77	1.25–2.52	1.61	1.11–2.34
Extended model 4 (*n* = 2515)				
EQ5D				
No problems	1.00	Reference	1.00	Reference
One or more problems	1.28	1.06–1.54	1.23	1.01–1.50

*Extended models are for individual current attitudes or status, controlling for the variables in the basic model.

**Table 3:** Informational needs and complementary and alternative medicine use.

Variables	Number of users/Number of patients	%	Multivariate analysis*	
			OR	95% CI
Needed information about the current status of my illness and its future course				
No	143/751	19.0	1.00	Reference
Yes	535/1910	28.0	1.61	1.29–2.02
Needed information about tests and treatments				
No	152/814	18.7	1.00	Reference
Yes	526/1847	28.5	1.68	1.35–2.09
Needed information about symptoms requiring a hospital visit				
No	187/897	20.9	1.00	Reference
Yes	491/1764	27.8	1.44	1.17–1.78
Needed an easy and accurate explanation of the benefits, side effects, and application of current medication				
No	240/1103	21.8	1.00	Reference
Yes	437/1556	28.1	1.33	1.10–1.62
Needed information about what I could do at home for my health (e.g., exercise)				
No	191/936	20.4	1.00	Reference
Yes	487/1725	28.2	1.49	1.21–1.83
Needed information about correct diet (foods to eat, foods to avoid)				
No	173/894	19.4	1.00	Reference
Yes	504/1765	28.6	1.63	1.32–2.02
Needed information about cancer-treating hospitals or clinics and physicians				
No	243/1196	20.3	1.00	Reference
Yes	435/1465	29.7	1.51	1.25–1.84
Needed information about governmental financial support for medical expenses				
No	137/708	19.4	1.00	Reference
Yes	541/1951	27.7	1.56	1.25–1.96
Needed information about hospice services				
No	476/1925	24.7	1.00	Reference
Yes	192/715	26.9	1.04	0.84–1.29

*Multivariate analysis was performed on individual informational need, controlling for the variables in the basic model (gender, age, monthly income, cancer type, metastasis, and time since diagnosis).
